# *trans*-Cinnamic acid-induced leaf expansion involves an auxin-independent component

**DOI:** 10.1080/19420889.2019.1605814

**Published:** 2019-04-19

**Authors:** Jasmina Kurepa, Jan A. Smalle

**Affiliations:** Plant Physiology, Biochemistry, Molecular Biology Program, Department of Plant and Soil Sciences, College of Agriculture, Food and Environment, University of Kentucky, Lexington, KY, USA

**Keywords:** Auxin, *trans*-cinnamic acid, phenylalanine ammonia-lyase, cinnamate-4-hydroxylase, leaf expansion

## Abstract

The phenylpropanoid pathway, the source of a large array of compounds with diverse functions, starts with the synthesis of *trans*-cinnamic acid (*t*-CA) that is converted by cinnamate-4-hydroxylase (C4H) into *p*-coumaric acid. We have recently shown that in Arabidopsis, exogenous *t*-CA promotes leaf growth by increasing cell expansion and that this response requires auxin signaling. We have also shown that cell expansion is increased in C4H loss-of-function mutants. Here we provide further evidence that leaf growth is enhanced by either *t-*CA or a *t-*CA derivative that accumulates upstream of C4H. We also show that this growth response pathway has two components: one that requires auxin signaling and another which employs a currently unknown mechanism.

The phenylpropanoid (PP) pathway generates a wide range of structurally and functionally different compounds whose importance for cell growth and development is widely recognized. [1] In the first two steps of this pathway, phenylalanine ammonia-lyase (PAL) deaminates L-phenylalanine to yield *trans*-cinnamic acid (*t*-CA) which is then converted into *p*-coumaric acid by cinnamate-4-hydroxylase (C4H). [1] We have recently shown that feeding with *t*-CA increases the growth rate and final size of Arabidopsis rosette leaves by increasing cell expansion. [2] Two previous studies on plant species other than Arabidopsis have also shown that *t-*CA treatment or increased *t-*CA accumulation leads to increased leaf growth, indicating that the growth promotion by *t-*CA is conserved within higher plants. [3,4] We have also shown that auxin insensitive mutants are resistant to *t-*CA-induced leaf expansion and concluded that *t-*CA-induced leaf growth requires an intact auxin signaling pathway. [2] To test weather *t-*CA itself acts to increase leaf cell expansion or serves as a precursor of a cell-size regulatory PP compound that is synthesized in a downstream branch, we analyzed the cell sizes in lines that have reduced PAL or C4H activity (Figure 1(a)) [2]. In transgenic plants with reduced PAL activity, caused by overexpression of the PAL-targeting ubiquitin ligase subunit KFB20, cells are smaller compared to the wild type [2] and consequently, the plants are dwarfed. [2,5] On the other hand, in *ref3* mutants, which have reduced C4H activity, [6] cells are larger than in the wild type. Because both *ref3* mutants and KFB20 overexpression plants are predicted to have a reduced carbon flow downstream of C4H, none of the compounds synthesized after the C4H step in the PP pathway can be this promoter of cell expansion and the active PP intermediate(s) is either a PAL product or its derivative. The exact chemical structure of this active compound(s) awaits further investigation.

**Figure 1.**
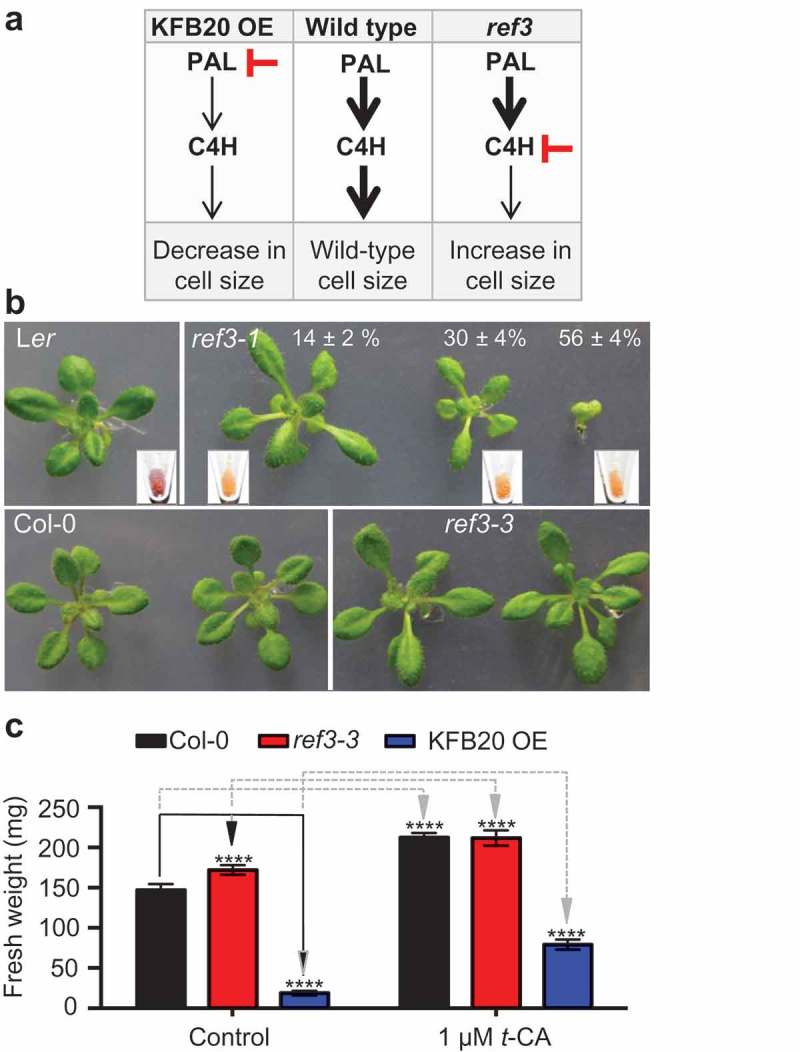
*t-*CA-induced rosette growth responses of *ref3* mutants. (a) Summary of previous findings that suggest that *t-*CA or a *t-*CA derivative promotes plant cell expansion. [2] PAL, phenylalanine ammonia-lyase; C4H, cinnamic acid 4-hydrolase; KFB20 OE, Kelch repeat F-box protein 20 overexpressor. (b) Rosettes of 20-day-old plants. The upper panel shows wild type plants (L*er*) and *ref3-1* plants grown on MS/2 (control) media. The frequencies of *ref3-1* rosette phenotypes are noted as percentages (n = 89). Insert shows the seeds of the progeny of each phenotypical class. The lower panel shows rosettes of the Col-0 wild type and *ref3-3* plants grown on control media. (c) Fresh weight of rosettes of Col-0, *ref3-3* and KFB20 OE plants grown on control and *t-*CA-supplemented medium for 20 days. Data are presented as mean ± SD (n ≥ 12 pools of 6 plants each). The significance of the difference between the mean fresh weight between lines grown on control media (black arrows) and between controls and the treated samples (dashed, gray arrows) is noted (****, P < 0.0001; two-way ANOVA with Tukey’s post-test).

To further explore the relationship between early PP pathway intermediates and leaf expansion, we analyzed the *t-*CA growth response of *ref3* mutants. We were unable to use the *ref3-1* mutant line for this purpose because when grown on control medium, different seedlings had different rosette growth rates, which separated them into three classes: wild-type-sized, semi-dwarfed and dwarfed, aberrant seedlings (Figure 1(b)). These three different classes of *ref3-1* plants retained their homozygous *ref3-1* genotype as evidenced by the pale seed color of their progeny (Figure 1(b)). [7] In addition, the progeny of each class retained the same pattern of developmental variability (data not shown). Because the *ref3-1* mutant is an auxin hypersensitive response mutant, [2] these developmental abnormalities and their incomplete penetrance are in agreement with the variability observed for some of the previously characterized auxin-related mutant lines. [8–10] The plants of the weaker C4H loss-of-function *ref3-3* mutant did not show any developmental variability. Surprisingly, *ref3-3* rosette leaves were larger than the wild-type leaves as illustrated by the measurements of rosette fresh weight that has been previously shown to be positively correlated with increased leaf size (Figure 1(b,c)). [2] This increase in rosette size stands in contrast to what was reported in the original *ref3* mutant study. [6] In that study, *ref3* plants were sown and grown on soil rather than on a sterile, sucrose-supplemented growth medium. [6] Since many of the products and intermediates of the PP pathway are involved in plant responses to the environment, [11,12] the discrepancy between the results of these two studies is potentially due to differential accumulation of specific PP compounds in the *ref3* mutants that are less permissive for growth under non-sterile conditions. As expected from our previous study, [2] *t-*CA treatments led to growth increases of 44.4 ± 4% in the wild-type plants and 420 ± 33% in the dwarfed KFB20 overexpression plants (Figure 1(c); [2]). *t*-CA feeding also led to an increase of the rosette size in *ref3-3* plants, but this response was attenuated compared to the other lines (23 ± 6%; Figure 1(c)). Because the rosette size increases caused by C4H loss of function and *t*-CA treatment were not additive (i.e., the *t*-CA-treated wild-type and *ref3-3* plants were of the same size), we concluded that the size increase in *ref3-3* plants is indeed related to the *t-*CA-induced effect on rosette growth. Moreover, the increased rosette size of *ref3-3* plants was in agreement with our earlier observation that treatments with low concentrations of the C4H inhibitor piperonylic acid (PA) lead to an increase in rosette leaf size. [2] Higher doses of PA as well as strong C4H loss-of- (e.g., *ref3-2*) mutations lead to a reduction in rosette size most likely because of the decreased biosynthesis of compounds downstream of C4H (e.g., lignins) which are needed for growth.

To better understand the role of auxin in *t*-CA-induced leaf expansion, we analyzed the *t-*CA and auxin effects separately and in combination. Treatments with either low concentrations of *t*-CA or IAA promoted leaf expansion. [2] When combined, *t-*CA and IAA led to an increase in leaf size in an additive manner: 1 µM *t*-CA led to a size increase of 40 ± 5%, 160 nM IAA led to a size increase of 18 ± 3% and when 1 µM *t*-CA and 160 nM of IAA were applied together, we observed a growth increase of 67 ± 4% (Figure 2(a)). Under our experimental conditions, 160 nM IAA was the maximum response dose: 320 nM IAA promoted growth but to a lesser extent than 160 nM IAA (Figure 2(a)). However, even at the highest tested IAA dose, the combined treatment caused a growth increase that was significantly stronger than that observed for the treatment with 160 nM IAA only (Figure 2(a)). This suggests an additive interaction between IAA- and *t*-CA-dependent leaf growth promotion mechanisms.

**Figure 2.**
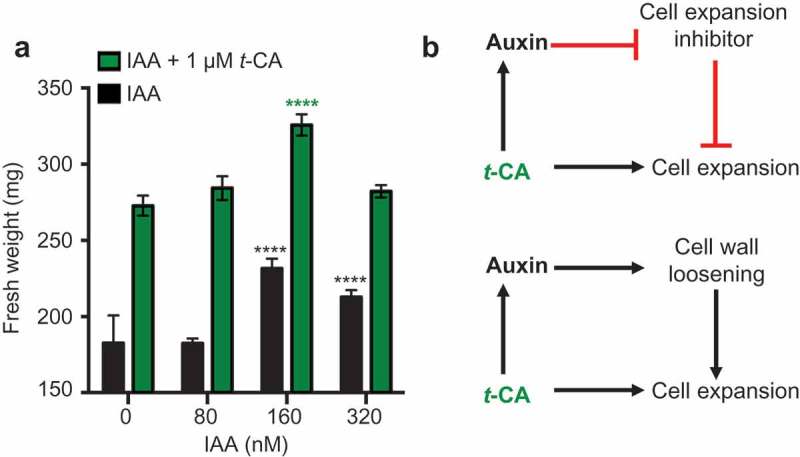
Comparative analysis of the effects of auxin and *t-*CA on rosette growth. (a) Effects of indole-3-acetic acid (IAA) on the fresh weight of rosettes from wild-type plants grown for 20 days on MS/2 media containing the denoted doses of *t*-CA and IAA. Data are presented as mean ± SD (n ≥ 12 pools of 8 plants each). All differences between the IAA and IAA/*t*-CA treated samples for each IAA concentration are significant at P < 0.0001 (not marked; two-way ANOVA with Sidak’s multiple comparisons test). The significance of the difference between the fresh weight of plants grown control media and on different IAA-supplemented media is shown in black and between different *t*-CA control and *t*-CA/IAA co-treated plants treatments is shown in green (****, P < 0.0001; two-way ANOVA with Sidak’s multiple comparisons test). (b) Hypothetical models for the additive mechanism of action of IAA and *t-*CA in leaf growth promotion.

In conclusion, *t*-CA-induced leaf expansion combines an auxin-dependent and an auxin-independent mechanism. In Figure 2(b), we offer two possible models to explain this dual *t*-CA effect on growth. In common to both models is the auxin-independent positive effect of *t-*CA on cell expansion. The second component of the first model is a *t-*CA-driven enhancement of auxin signaling that is needed to inhibit an auxin-dependent repressor of cell expansion. The second point of the second model is cell wall loosening, a well-known auxin effect. [13] According to this model, the *t-*CA-dependent cell expansion can commence only if *t-*CA in parallel enhances auxin-dependent cell wall loosening. Regardless of which of these models is true, the fact that the auxin-dependent and *t-*CA-dependent effects are additive, suggests that plants have an intricate and multifaceted *t*-CA-governed mechanism that affects growth.

## Materials and methods

The wild-type lines used were Columbia (Col-0) and Landsberg *erecta* (L*er*) which are the backgrounds for the *ref3-3* and *ref3-1* mutants, respectively. [6] Both *ref3* mutant lines were obtained from the ABRC Seed Stock Center. All plants were grown in sterile conditions on half-strength Murashige and Skoog medium (pH 5.7) containing 1% sucrose and 0.8% PhytoAgar (MS/2 medium). The following chemicals were used for treatments: indole-3-acetic acid (IAA; Sigma) and *trans*-cinnamic acid (*t-*CA; Sigma). All rosette growth response analyses were done as previously described. [2] The descriptive statistics, plotting and hypothesis testing were done using Prism 6 software (GraphPad Software Inc). All data are presented as means ± SD of at least three independent experiments. When means of more than two samples were compared, we used ANOVA followed by post-test to find a significant difference between pairs of means. The significance levels, indicated by asterisks in the figures, illustrate the results of the post-test.

## References

[1] Vogt T. Phenylpropanoid biosynthesis. Mol Plant. 2010;3:2–20. 20035037 10.1093/mp/ssp106

[2] Kurepa J, Shull TE, Karunadasa SS, et al Modulation of auxin and cytokinin responses by early steps of the phenylpropanoid pathway. BMC Plant Biol. 2018;18:278. 30419822 10.1186/s12870-018-1477-0 PMC6233370

[3] Talaat IM, Balbaa LK Physiological response of sweet basil plants (*Ocimum basilicum* L.) to putrescine and *trans*-cinnamic acid. Am Eurasian J Agric Environ Sci. 2010;8:438–445.

[4] Orlova I, Marshall-Colón A, Schnepp J, et al Reduction of benzenoid synthesis in petunia flowers reveals multiple pathways to benzoic acid and enhancement in auxin transport. Plant Cell. 2006;18:3458–3475. 17194766 10.1105/tpc.106.046227 PMC1785411

[5] Zhang X, Gou M, Liu CJ Arabidopsis Kelch repeat F-box proteins regulate phenylpropanoid biosynthesis via controlling the turnover of phenylalanine ammonia-lyase. Plant Cell. 2013;25:4994–5010. 24363316 10.1105/tpc.113.119644 PMC3904001

[6] Schilmiller AL, Stout J, Weng JK, et al Mutations in the cinnamate 4-hydroxylase gene impact metabolism, growth and development in Arabidopsis. Plant J. 2009;60:771–782. 19682296 10.1111/j.1365-313X.2009.03996.x

[7] Ruegger M, Chapple C Mutations that reduce sinapoylmalate accumulation in *Arabidopsis thaliana* define loci with diverse roles in phenylpropanoid metabolism. Genetics. 2001;159:1741–1749. 11779811 10.1093/genetics/159.4.1741 PMC1461910

[8] Bennett SRM, Alvarez J, Bossinger G, et al Morphogenesis in *pinoid* mutants of *Arabidopsis thaliana*. Plant J. 1995;8:505–520.

[9] Lie C, Kelsom C, Wu X WOX2 and STIMPY-LIKE/WOX8 promote cotyledon boundary formation in Arabidopsis. Plant J. 2012;72:674–682. 22827849 10.1111/j.1365-313X.2012.05113.x

[10] Ploense SE, Wu MF, Nagpal P, et al A gain-of-function mutation in IAA18 alters Arabidopsis embryonic apical patterning. Development. 2009;136:1509–1517. 19363152 10.1242/dev.025932 PMC2674258

[11] Dixon RA, Achnine L, Kota P, et al The phenylpropanoid pathway and plant defence - a genomics perspective. Mol Plant Pathol. 2002;3:371–390. 20569344 10.1046/j.1364-3703.2002.00131.x

[12] Lee HI, Leon J, Raskin I Biosynthesis and metabolism of salicylic acid. Proc Natl Acad Sci USA. 1995;92:4076–4079. 11607533 10.1073/pnas.92.10.4076 PMC41889

[13] Majda M, Robert S The role of auxin in cell wall expansion. Int J Mol Sci. 2018;19:951. 10.3390/ijms19040951 PMC5979272 29565829

